# Bibliometric analysis of the global research development of bone metastases in prostate cancer: A 22-year study

**DOI:** 10.3389/fonc.2022.947445

**Published:** 2022-09-27

**Authors:** Yongming Chen, Chen Tang, Zefeng Shen, Shengmeng Peng, Wanhua Wu, Zhen Lei, Jie Zhou, Lingfeng Li, Yiming Lai, Hai Huang, Zhenghui Guo

**Affiliations:** ^1^ Department of Urology, Sun Yat-sen Memorial Hospital, Sun Yat-sen University, Guangzhou, China; ^2^ Guangdong Provincial Key Laboratory of Malignant Tumor Epigenetics and Gene Regulation, Sun Yat-sen Memorial Hospital, Sun Yat-sen University, Guangzhou, China; ^3^ Guangdong Provincial Clinical Research Center for Urological Diseases, Sun Yat-Sen Memorial Hospital, Sun Yat-Sen University, Guangzhou, China

**Keywords:** prostate cancer, bone metastases, bibliometric, VOSviewer, Citespace

## Abstract

**Background:**

Prostate cancer (PCa) is the second most diagnosed cancer in men. Most PCa-related deaths result from metastatic disease. Metastases occur most often in the bones (90%). However, the current treatments for bone metastases in PCa are not very effective. Here we present an overview of the current research situation of bone metastases in PCa, focusing on hotspots and trends.

**Methods:**

We searched the Web of Science Core Collection database for publications related to bone metastases in PCa published between 1999 and 2021. We used VOSviewer, CiteSpace, and a bibliometric online platform to perform a bibliometric analysis of countries, institutions, authors, journals, references, and keywords.

**Results:**

A total of 4,832 related articles were included in the present study. The USA published the most articles in the field, followed by China and England. The University of Texas MD Anderson Cancer Center is the leading institution in the research field of bone metastases in PCa. Saad F, from Canada, has made great achievements in this area by publishing 91 related articles. *Prostate* is the journal which published most related articles, and Mundy GR, 2002, *Nat Rev Cancer*, is the most cited article in this field. Furthermore, the analysis of author keywords can be divided into five clusters: (1) diagnosis of PCa, (2) mechanism of bone metastasis, (3) drug treatments of bone metastases, (4) radiotherapy of bone metastases, and (5) treatments and prognosis of PCa.

**Conclusions:**

mCRPC has been the hottest topic in PCa in recent years. CT is the most common diagnostic method for bone metastases. Enzalutamide and radium-223, as important treatments for bone metastases in PCa, bring about widespread attention. Furthermore, the researchers focus on the tumor microenvironment and biomarkers to explore the mechanism and the therapeutic targets of bone metastases in PCa.

## Introduction

Prostate cancer (PCa) is the second most diagnosed cancer type in men (1). The survival rate of patients with localized or regional PCa is relatively good, with a 5-year survival rate of 100%. However, once metastases develop, prognosis is poor, and the 5-year survival rate declines to 29.8% (2). Among the metastatic sites of PCa, bone, especially the axial skeleton, is the most frequently occurring metastatic site, accounting for 90% (3, 4). Bone metastases (BM) can be characterized morphologically as osteolytic, osteoblastic, or mixed. BM in PCa are predominantly osteoblastic (5). Skeletal-related events (SREs) are the major complications of BM and can affect the prognosis of BM patients. SREs include pathological fractures, spinal cord compression, hypercalcemia, the need for radiotherapy to relieve bone pain or reduce structural bone damage, and surgery to bone to prevent or repair a fracture (6).

As yet, the exact mechanisms causing BM remain unknown. It is known that the bone marrow has a high blood flow, and PCa cells release adhesive molecules that bind them to marrow stromal cells and bone matrix (7, 8). Additionally, bone stores a lot of growth factors, including fibroblast growth factors, insulin-like growth factors I and II, transforming growth factor β, platelet-derived growth factors, and bone morphogenetic proteins, and calcium (9). Bone will release these growth factors to provide a fertile ground for the growth of tumor cells during bone resorption (10). This “seed-and-soil hypothesis”, proposed by Stephan Paget in 1889 (11), can roughly explain the mechanism of the process of BM, but more specific research is required.

The current treatments of BM are mainly aimed at preventing disease progression and symptom palliation but not cure—for example, bone-targeted agents, such as bisphosphonates and denosumab, have been proved to improve bone structure and quality to minimize the risk of skeletal morbidity (12–14). The clinical burden on patients and healthcare systems is very large due to the high incidence of BM. According to the World Health Organization (WHO), there were 1.3 million PCa patients, and 359,000 of them died of cancer in 2018 (1). Therefore, it is critical to develop strategies to prevent and treat BM and improve the quality of life and survival of BM patients.

Bibliometric analysis is an information visualization method to identify and summarize the frontiers or hot spots in a certain area. The quantification of literature in this field is based on mathematical and statistical methods through the analysis of literature and metrological characteristics. Moreover, we can compare the research status among various institutions, authors, or journals, evaluate the latest cutting-edge research, understand the scientific articles, and visualize their trends through this method.

We aimed to show the developments and trends in the research field of BM in PCa in the past 22 years by bibliometric analysis. Moreover, our study provides an overview of the research of BM in PCa that can serve as a reference for researchers.

## Methods

### Database

The data source of our research is the Science Citation Index Expanded (SCI-Expanded) of Clarivate Analytics’ Web of Science Core Collection (WoSCC). The WoSCC is a widely used database for bibliometric studies which contains publications from nearly 9,000 high-impact journals.

### Search strategy

A search for publications related to PCa and BM was performed on April 25, 2022. The search query was formulated as follows: topic = “(prostate OR prostatic) NEAR/1 (cancer OR tumor OR tumor OR oncology OR neoplasm OR carcinoma)” AND “bone metastasis OR bone metastases” AND “publication date = (January 1, 1999–December 31, 2021)”. The search results were downloaded in plain text format and in the tab separator format of “Full Records and cited References”. Furthermore, the publication types were limited to original articles and reviews, and the language was limited to English.

### Data analysis and visualization

To ensure the accuracy of data and the reliability of the research, two researchers independently downloaded and analyzed the data. Co-authorship, co-occurrence, and co-citation analysis are the most significant indicators in bibliometric analysis. Co-authorship analysis is conducted to analyze the relationships among authors, countries, or institutions. Co-occurrence analysis is a quantitative method to analyze the most frequently appearing items in articles. A co-citation analysis was conducted by comparing the ranked results with the co-citation score.

Microsoft Excel 2019 was used to analyze the annual publications and generate the line graph and to produce the tables of top-cited or productive countries/regions, publications, journals, and authors.

VOSviewer (version 1.6.18), CiteSpace (version 5.8.R3), and a widely used online platform for bibliometric analysis were used to visualize the data. With VOSviewer, one can map network data based on computer programs. It can be used to create a co-authorship, keyword co-occurrence, citation, bibliographic coupling, or co-citation map based on bibliographic data. Furthermore, The VOSviewer shows not only how the subjects are related to each other but also how far away in time they are in different colors. Therefore, we can use this visualization to predict future research hotspots. CiteSpace is mainly used to analyze the potential knowledge contained in the scientific literature and for data visualization. We used CiteSpace to analyze the co-authorship of institution and author, the co-authorship of reference and journal, and the co-citation of authors. We can learn about current and even future research hotspots from the reference’s citation bursts. In addition, we generated a dual map overlay of journals by CiteSpace. The online platform for bibliometric analysis was applied to analyze the country/region’s publications and co-authorship.

### Research ethics

All data were obtained from the public database. As a result, there was no need to seek ethics approval.

## Results

### Data collection and the trend of publication outputs

**Figure 1:**
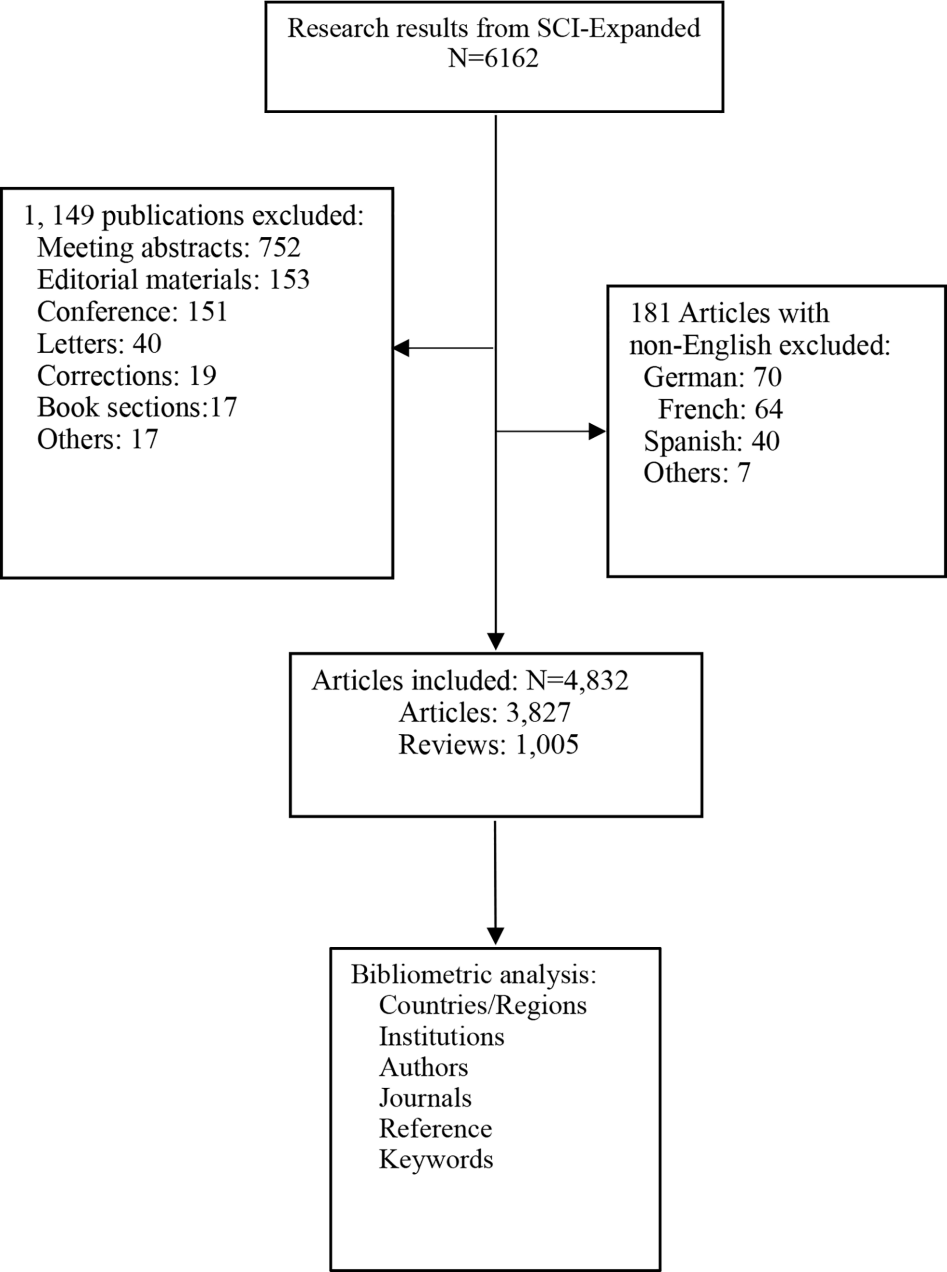
Flow chart of articles selection.

We obtained 6,162 related publications from the WoSCC database (**Figure 1**). However, 1,149 papers of them, including 752 meeting abstracts, 153 editorial materials, 151 conferences, 40 letters, 19 corrections, 17 book sections, and 17 other types of publications, were excluded. Moreover, 181 articles were also excluded because they were not written in English. Finally, a total of 4,832 articles, including 3,827 original articles and 1, 005 reviews, were included.

**Figure 2:**
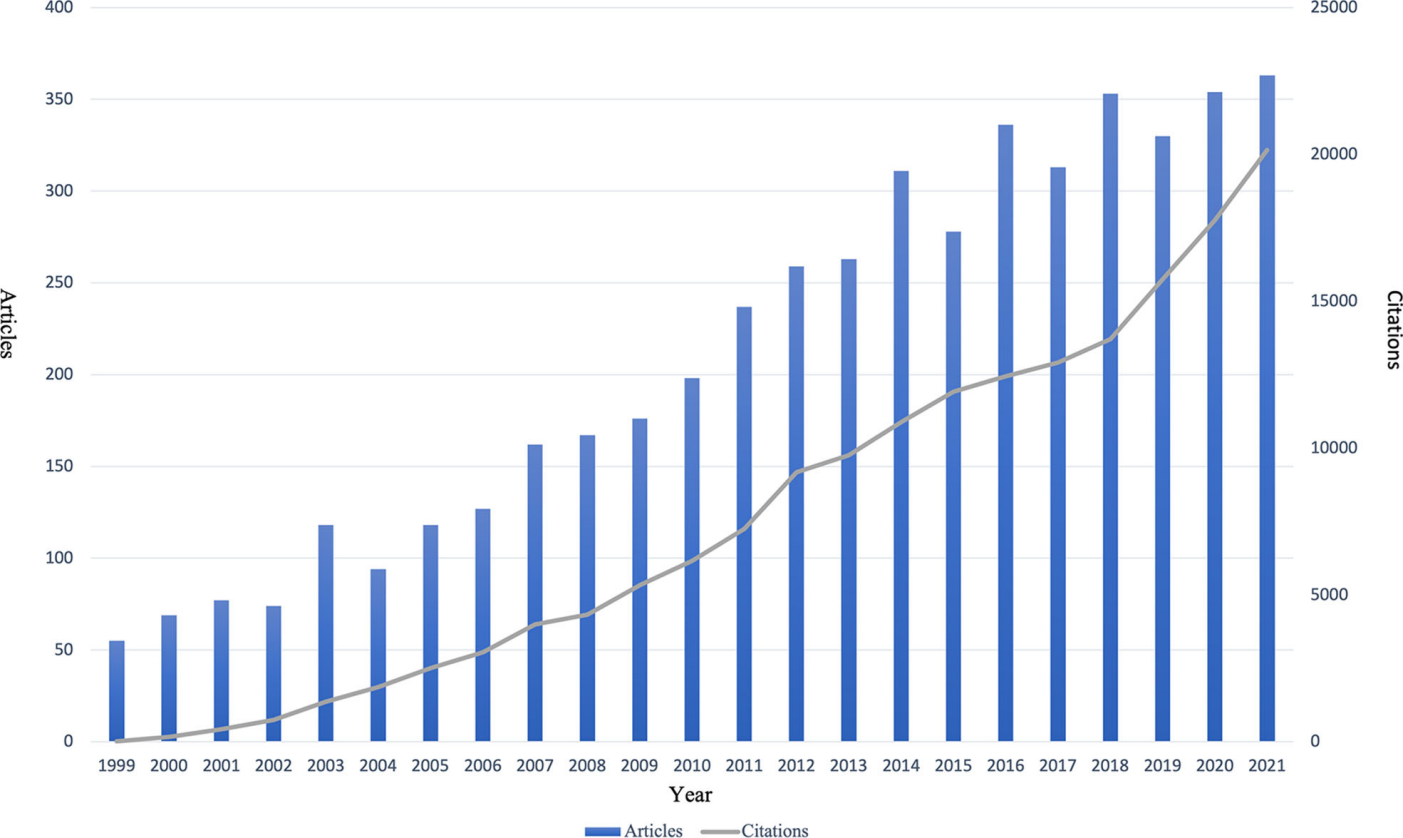
Quantitative analysis of the published articles per year.

The quantitative analysis of published articles about BM in PCa is shown in **Figure 2**. We can see that the number of published articles increased from 1999 to 2016, and the increase was fastest from 2004 to 2014. However, the number of published articles increased less rapidly from 2017 to 2021, when it remained stable at a level of 300 to 400 articles per year.

### Analysis of published papers per country/region

**Figure 3:**
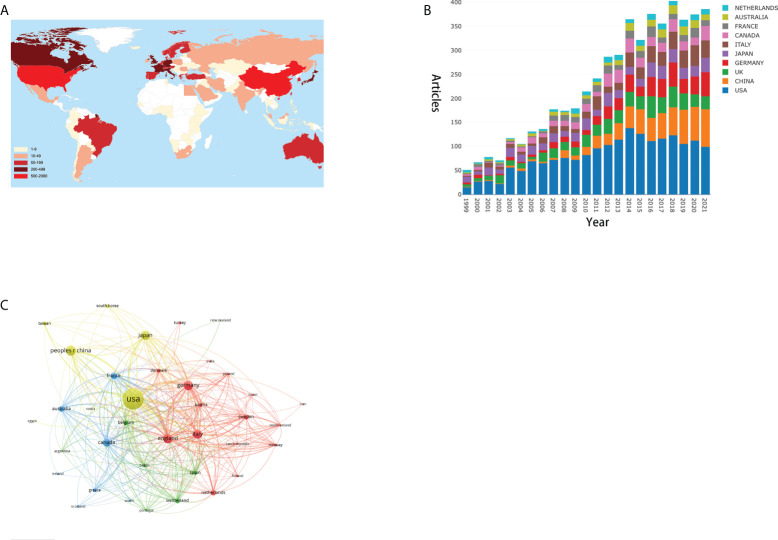
**(A)** Total articles per country/region. A different color indicates a different output. **(B)** Bar graph of the top 10 productive countries/regions. **(C)** Correlations among the countries/regions with more than 15 articles. The graph was generated by VOSviewer. Line thickness indicates the citation strength.

In total, 4,832 articles were published by 4,872 organizations in 92 countries (**Figure 3A**). The USA published the most articles in this period (1,890, 39.1%) (**Figure 3B**; **Table 1**). China, England, Germany, Japan, and Italy also published more than 400 articles each. In addition, USA’s total number of citations (98,773) and H-index (142) both ranked first. The country collaboration network is shown in **Figure 3C**. **Figure 3C**, which was generated by VOSviewer. In total, 37 countries that published more than 15 articles were included. The thickness of the line demonstrates the strength of cooperation among countries (named as total link strength, TLS). The top five TLSs were the USA, England, Germany, Canada, and Italy.

**Table 1:** Top 10 productive countries/regions associated with the bone metastases in prostate cancer.

Rank	Country	Documents	Percentage	TC	AAC	H-index
1	USA	1,890	39.11%	98,773	52.26	142
2	China	535	11.07%	10,733	20.06	51
3	England	473	9.79%	28,719	60.72	82
4	Germany	453	9.38%	20,014	44.18	76
5	Japan	441	9.13%	14,353	32.55	55
6	Italy	428	8.86%	16,017	37.42	66
7	Canada	343	7.10%	19,385	56.52	70
8	France	234	4.84%	16,162	69.07	69
9	Australia	197	4.08%	14,220	72.18	57
10	Netherlands	180	3.73%	8,793	48.85	47

BM, bone metastases; PCa, prostate cancer; TC, total citations; AAC, average article citations.

### Top productive institutions

**Figure 4:**
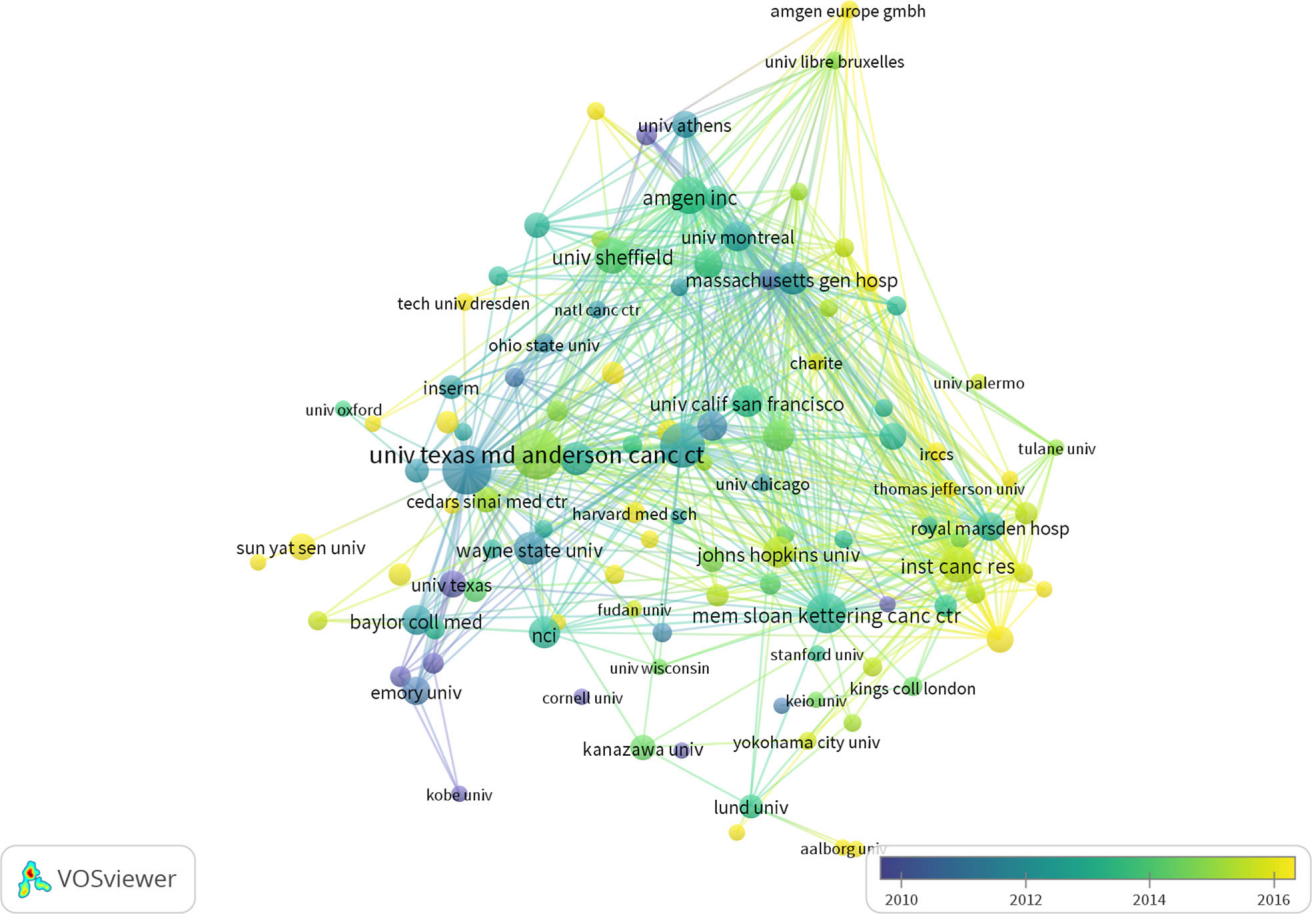
Overlay visualization map of the institutions with more than 20 articles.

In total, 4,872 institutions produced related articles. The most productive institutions were from the USA. As shown in **Table 2**, among the top 10 productive institutions, eight come from the USA and two are from the UK. The University of Texas MD Anderson Cancer Center (145) was the most productive institution, followed by the University of Michigan (139), the University of Washington (120), the Memorial Sloan-Kettering Cancer Center (97), and Amgen Inc. (89). We used VOSviewer to analyze the cooperation among institutions (**Figure 4**). With a threshold of ≥20 articles published, 110 institutions were included, and the top five TLSs were Amgen Inc., Massachusetts General Hospital, the University of Michigan, Inst Cancer Research, and the University of Washington.

**Table 2:** Top 10 productive institutions in publications related to the research on bone metastases in prostate cancer.

Rank	Institution	Countries/regions	Articles	Citations	TLS
1	University of Texas MD Anderson Cancer Center	USA	145	6,477	2,827
2	University of Michigan	USA	139	8,902	3,194
3	University of Washington	USA	120	6,640	2,891
4	Memorial Sloan-Kettering Cancer Center	USA	97	7,583	2,655
5	Amgen Inc	USA	89	7,510	3,813
6	University of Sheffield	UK	81	4,509	2,544
7	Inst Cancer Research	UK	79	5,278	3,119
8	California State University, Los Angeles	USA	69	3,486	974
9	Massachusetts General Hospital	USA	68	8,262	3,297
10	Wayne State University	USA	68	3,823	965

TLS, total link strength.

### Analysis of authors and co-cited authors

**Figure 5:**
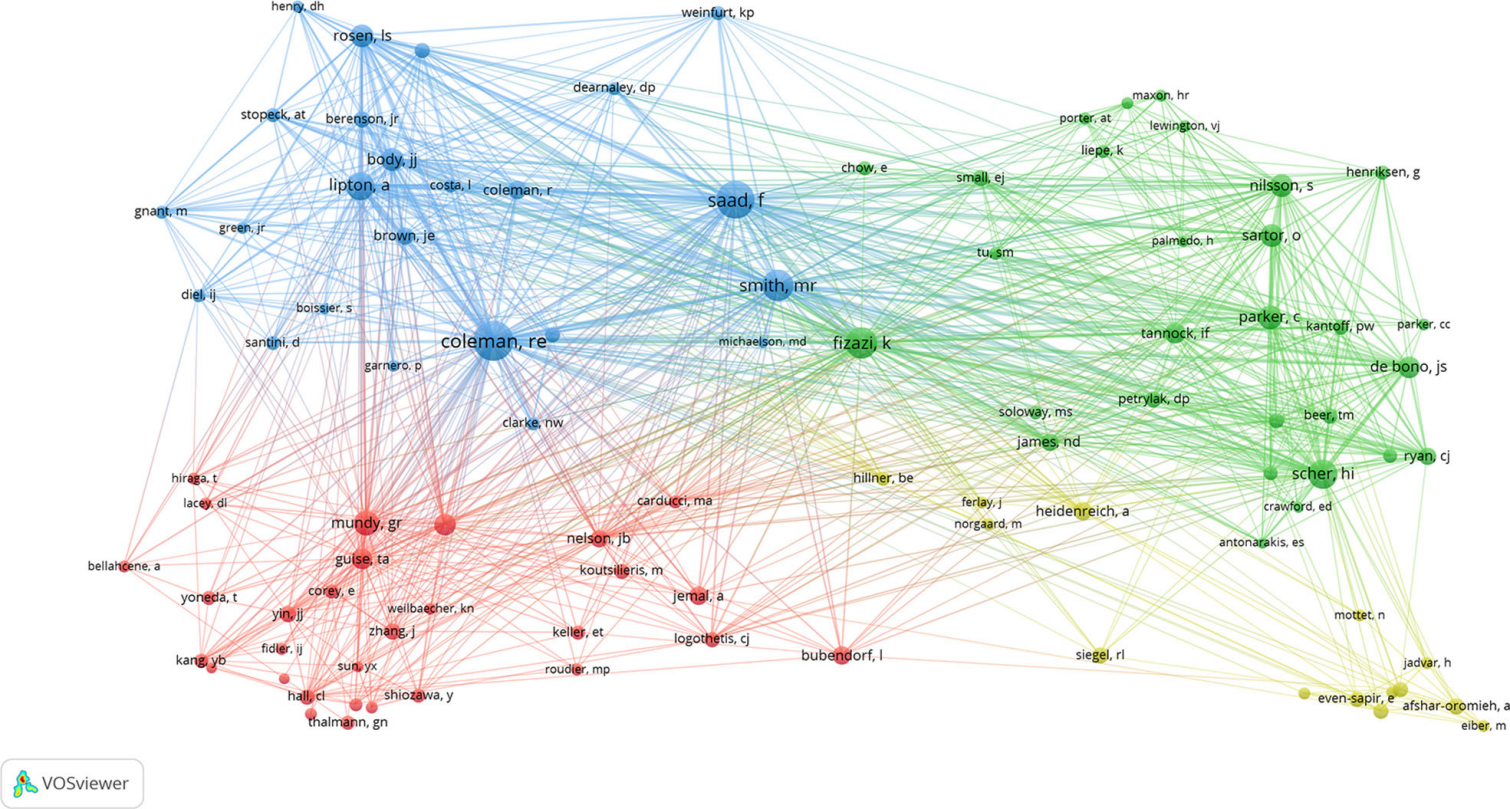
Visualization map of co-cited authors generated by VOSviewer.

In total, 23,495 authors and 64,811 co-cited authors were identified. The top 10 productive authors and the top 10 co-cited authors are shown in **Table 3**. Saad F (91), Logothetis CJ (50), Chung LWK (49), Fizazi K (47), and Keller ET (47) were the top five productive authors in the past 22 years. Saad F was the most cited author with a total number of 7,703 citations, and his H-index was the highest among the authors. However, Smith MR’s average article citations (AAC) ranked first (138.74). Moreover, the top five co-cited authors were Coleman RE (1,813), Saad F (1,598), Fizazi K (1,148), Smith MR (1,131), and Scher HI (988). The cooperation among co-cited authors who were cited more than 150 times is shown in **Figure 5**.

**Table 3:** The 10 most productive authors and the top 10 co-cited authors with the highest number of citations.

Rank	Author	Country	Documents	Citation	Average article citations	H-Index	Co-cited author	Country	Total citations
1	Saad, F	Canada	91	7,703	84.65	39	Coleman, RE	UK	1,813
2	Logothetis, CJ	USA	50	3,132	62.64	29	Saad, F	USA	1,598
3	Chung, LWK	USA	49	3,049	62.22	33	Fizazi, K	UK	1,148
4	Fizazi, K	UK	47	6,327	134.62	32	Smith, MR	USA	1,131
5	Keller, ET	USA	47	2,728	58.04	30	Scher, HI	USA	988
6	Pienta, KJ	USA	47	2,890	61.49	28	Lipton, A	USA	947
7	Smith, MR	USA	46	6,382	138.74	34	Mundy, GR	USA	689
8	Coleman, RE	UK	45	3,478	77.28	33	Parker, C	UK	684
9	Lin, SH	USA	43	1,439	33.47	23	Body, JJ	Belgium	625
10	Sartor, O	UK	43	3,703	114	25	Nilsson, S	Sweden	625

### Analysis of the top publishing journals

**Figure 6:**
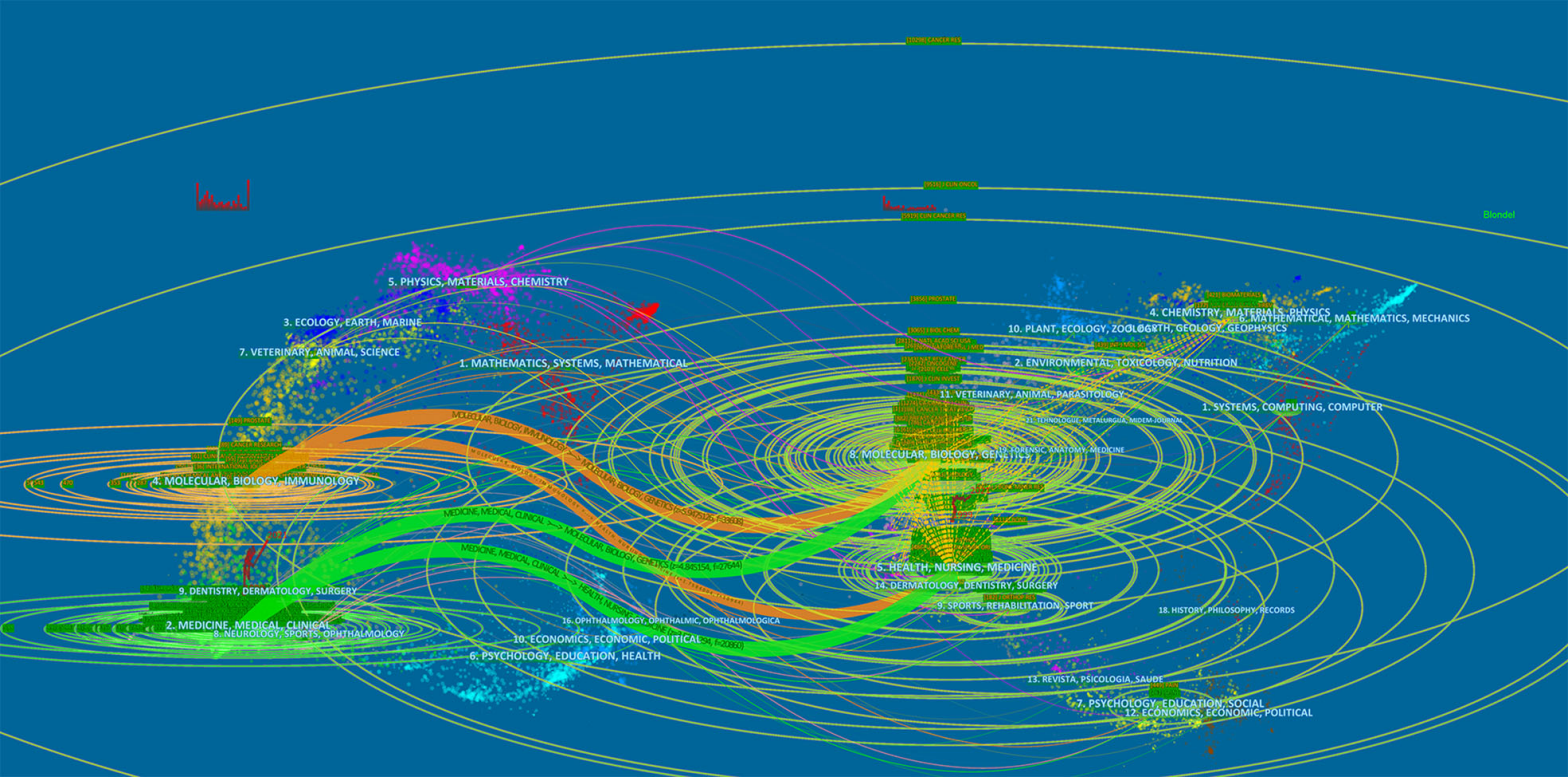
A dual map overlap of journals publishing articles on bone metastases in prostate cancer generated by CiteSpace.

A total of 873 journals have published related articles. The top 10 publishing journals are shown in **Table 4**. *Prostate* (149), *European Journal of Nuclear Medicine and Molecular Imaging* (92), *Cancer Research* (89), *Clinical Cancer Research* (80), and *Anticancer Research* (74) were the most contributing journals. Among the top 10 journals, 60% were in JCR Q1, and their impact factors were relatively high, with 70% being higher than 5.0. Besides this, *Cancer Research* had both the highest number of citations and the highest H-index. A dual map reflecting the relationship between citing and cited journals is shown in **Figure 6**. There are four citation pathways in the dual map. The citing papers were classified into two research areas: (1) medicine, medical, and clinical and (2) molecular biology and immunology. Furthermore, the cited papers were also classified into two fields: (1) health, nursing, and medicine and (2) molecular biology and genetics.

**Table 4:** The top 10 journals with the most articles associated with bone metastases in prostate cancer.

Rank	Journal	Country	IF (2021)	JCR (2021)	Articles	Citations	ACC	H-Index
1	*Prostate*	USA	4.104	Q3	149	5,927	39.78	43
2	*European Journal of Nuclear Medicine and Molecular Imaging*	Germany	9.236	Q1	92	4,578	49.76	39
3	*Cancer Research*	USA	12.701	Q1	89	8,455	95.00	53
4	*Clinical Cancer Research*	USA	12.531	Q1	80	6,001	75.01	46
5	*Anticancer Research*	Greece	2.480	Q4	74	1,080	14.59	18
6	*Journal Of Nuclear Medicine*	USA	10.057	Q1	73	4,252	58.23	35
7	*Plos One*	USA	3.240	Q2	72	2,178	30.25	24
8	*European Urology*	Netherlands	20.096	Q1	66	4,367	66.17	41
9	*Bju International*	UK	5.588	Q1	62	1,800	29.03	27
10	*Clinical & Experimental Metastasis*	Netherlands	5.150	Q3	61	2,236	36.66	26

IF, impact factor.

### Analysis of the top co-cited references and the references with the strongest citation bursts

**Figure 7:**
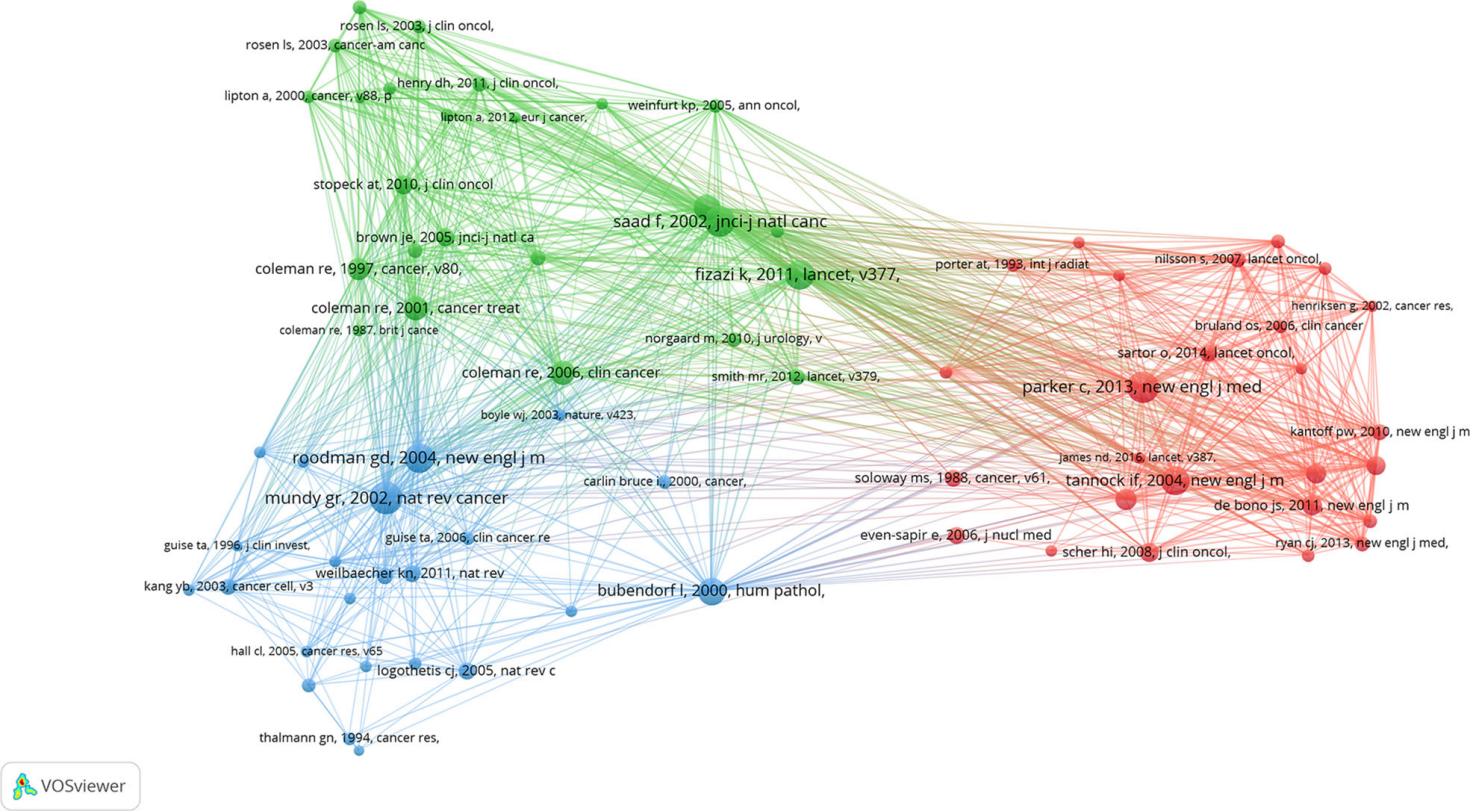
Co-cited reference collaboration network visualized by VOSviewer.

**Figure 8:**
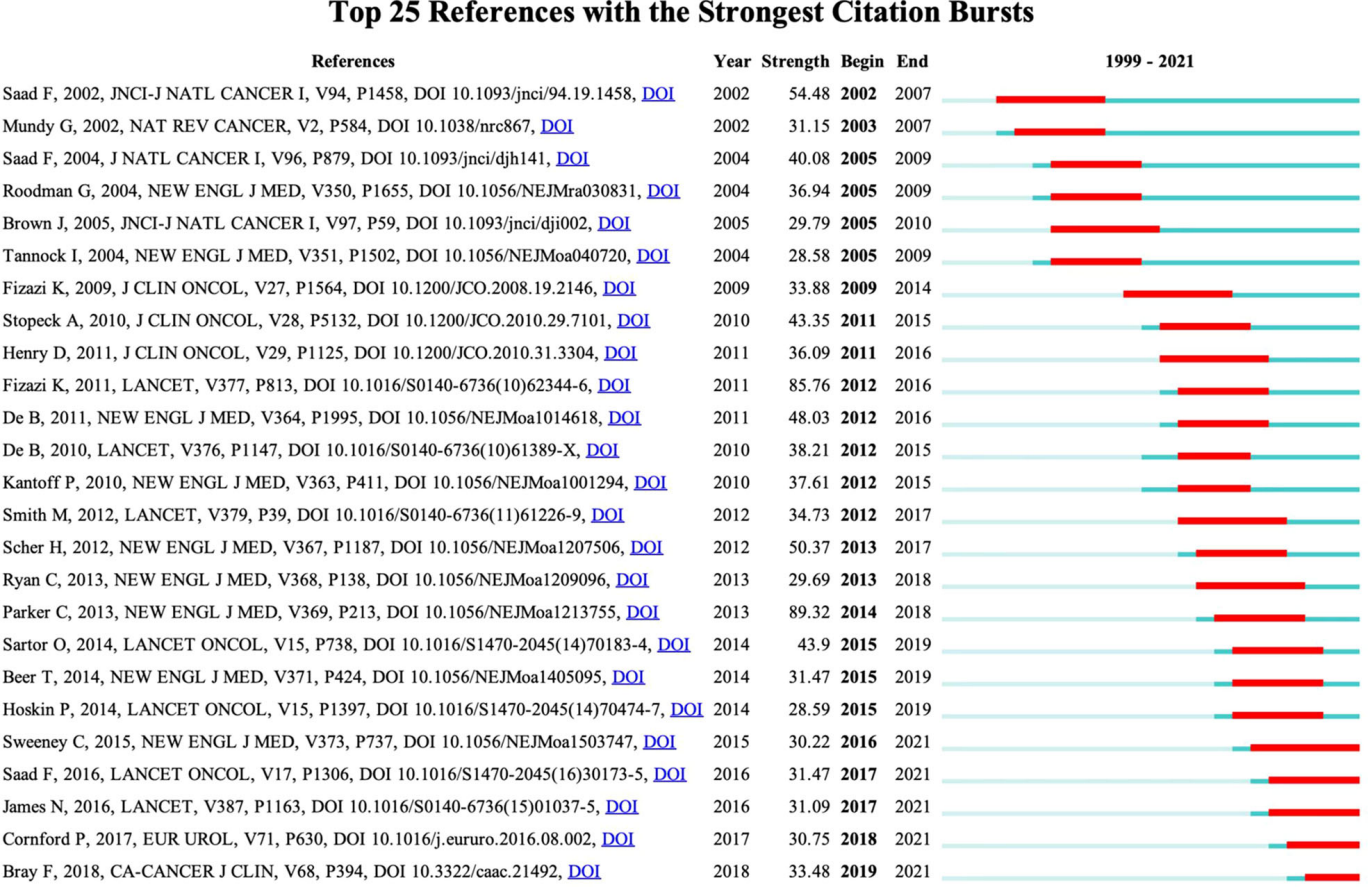
Visualization map of top 25 references with the strongest citation bursts from 1999 to 2021 generated by CiteSpace.

We used VOSviewer to analyze the co-cited references. The threshold was set as 100 cited times, and 74 references were included. The co-cited collaboration network is shown in **Figure 7**. The top 10 co-cited references are shown in **Table 5**. The top five co-cited references were Mundy GR, 2002, *Nat Rev Cancer* (464), Parker C, 2013, *New Engl J Med* (454), Saad F, 2002, *Jnci-J Natl Cancer I* (430), Roodman GD, 2004, *New Engl J Med* (406), and Fizazi K, 2011, *Lancet* (405). Interestingly, these top co-cited references were published relatively long ago, and most of them were published in journals with a high impact factor, which indicates that they are authoritative articles in the research field of BM in PCa. The top 25 references with the strongest citation bursts are shown in **Figure 8**. The first citation burst started in 2002, and there have been a lot of explosions since 2011. Notably, many citation bursts are still ongoing, which means that the field of BM in PCa is still a research hotspot.

**Table 5:** Top 10 co-cited references concerning the research of bone metastases in prostate cancer.

Rank	Title	Journals	Authors	Year	Citations
1	Metastasis to bone: causes, consequences and therapeutic opportunities	*Nat Rev Cancer*	Mundy GR, et al.	2002	464
2	Alpha emitter radium-223 and survival in metastatic prostate cancer	*New Engl J Med*	Parker C, et al.	2013	454
3	A randomized, placebo-controlled trial of zoledronic acid in patients with hormone-refractory metastatic prostate carcinoma	*Journal Of the National Cancer Institute*	Saad F, et al.	2002	430
4	Mechanisms of bone metastasis	*New Engl J Med*	Roodman GD, et al.	2004	406
5	Denosumab *versus* zoledronic acid for treatment of bone metastases in men with castration-resistant prostate cancer: a randomised, double-blind study	*Lancet*	Fizazi K, et al.	2011	405
6	Docetaxel plus prednisone or mitoxantrone plus prednisone for advanced prostate cancer	*New Engl J Med*	Tannock IF, et al.	2004	394
7	Metastatic patterns of prostate cancer: an autopsy study of 1,589 patients	*Hum Pathol*	Bubendorf L, et al.	2000	368
8	Long-term efficacy of zoledronic acid for the prevention of skeletal complications in patients with metastatic hormone-refractory prostate cancer	*J Natl Cancer Inst*	Saad F, et al.	2004	339
9	Metastatic bone disease: clinical features, pathophysiology and treatment strategies	*Cancer Treat Rev*	Coleman RE, et al.	2001	316
10	Clinical features of metastatic bone disease and risk of skeletal morbidity	*Clin Cancer Res*	Coleman RE, et al.	2006	316

### Analysis of the top co-occurrences of keywords

**Figure 9:**
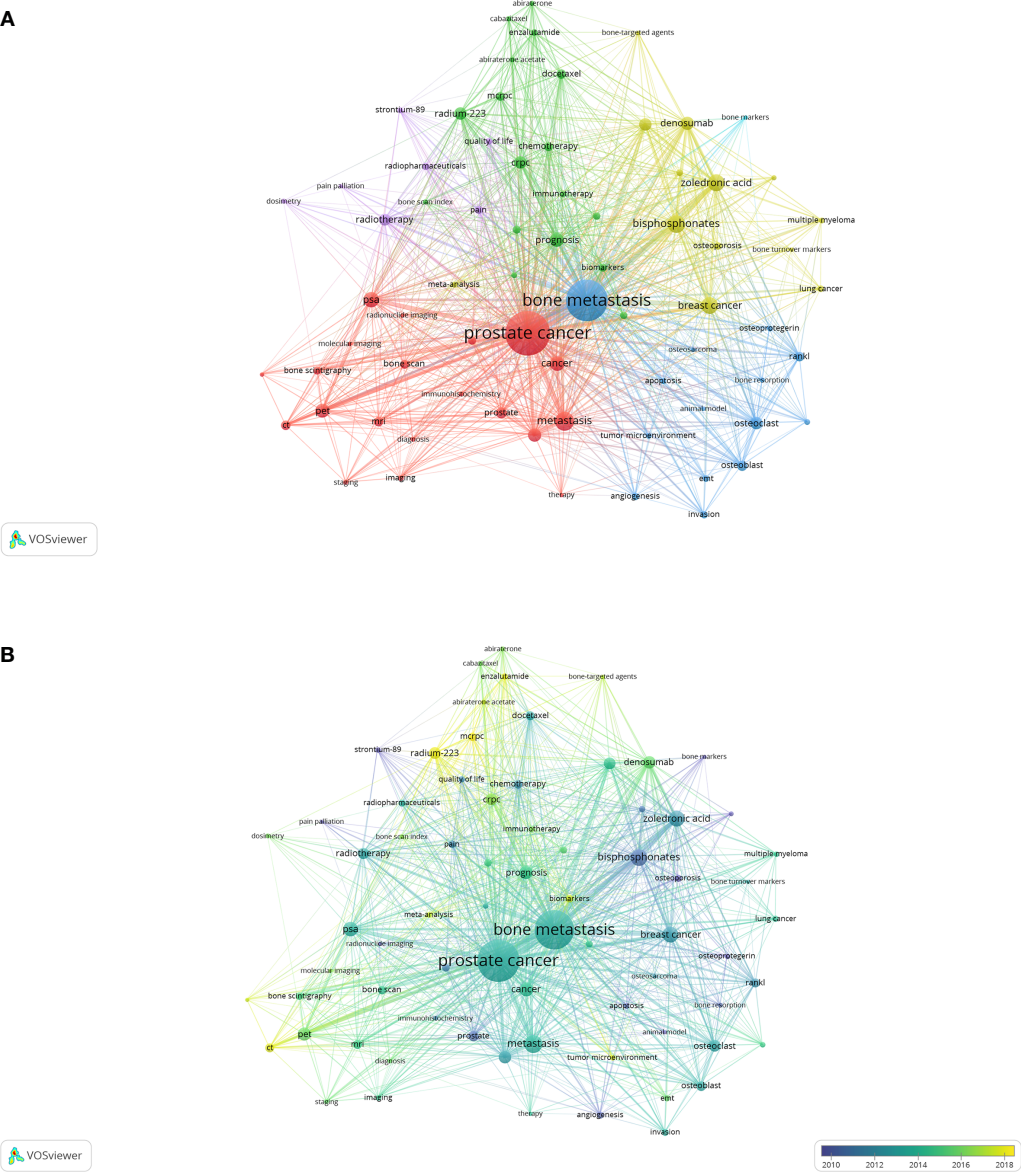
Networks generated by VOSviewer. **(A)** Visualization map of the author keywords with more than 20 occurrences. **(B)** Overlay visualization map of the author keywords with more than 20 occurrences.

The analysis of the co-occurrences of keywords was performed by VOSviewer. A total of 6,150 author keywords were identified, and 72 keyword co-occurrences were found more than 20 times; their relationship network is shown in **Figure 9**. The network displays five colors, representing five clusters, and the thickness of lines reflects the relationship between keywords. The keyword “prostate cancer”, with a TLS of 1,867, is located in the central position of the red cluster, which concentrates on the diagnosis of PCa with the keywords “diagnosis”, “immunohistochemistry”, “staging”, and so on. The blue cluster, with the central keyword “bone metastasis”, is focused on the mechanism of BM with the keywords “tumor microenvironment”, “angiogenesis”, “EMT”, “osteoblast”, and so on. The yellow cluster concentrates on the drug treatment of BM, the purple cluster reflects the radiotherapy of BM, and the green cluster shows the treatments and the prognosis of PCa. Furthermore, the overlay visualization of keywords, which shows the relationship of keywords and time, is shown in **Figure 9B**. The top 20 occurrences of keywords are shown in **Table 6**. Interestingly, “breast cancer” ranks fifth with 253 occurrences.

**Table 6:** Top 20 author keywords with the most occurrences in the included articles.

Keyword	Occurrences	Total link strength (TLS)	Keyword	Occurrences	TLS
Prostate cancer	1,703	1,867	Bone	165	348
Bone metastasis	1,535	1,890	Denosumab	164	380
Metastasis	327	513	Osteoclast	140	280
Bisphosphonates	279	533	Radium-223	137	197
Breast cancer	253	470	SRE	135	299
Zoledronic acid	248	507	CRPC	134	154
PSA	206	309	Radiotherapy	126	172
Cancer	194	301	Prostate	111	176
Prognosis	182	274	Osteoblast	98	205
PET	174	277	MRI	83	136

PSA, prostate-specific antigen; PET, positron emission tomography; SRE, skeletal-related events; CRPC, castration-resistant prostate cancer; MRI, magnetic resonance imaging.

## Discussion

To our knowledge, this is the first bibliometric analysis about BM in PCa. We analyzed the research situation and provided a reference to researchers in the field. According to data from the WoSCC database on April 25, 2022, a total of 4,832 articles associated with BM in PCa have been published between 1999 and 2021. The number of publications is an essential indicator of the trends in the research area (15). The number of publications per year gradually increased from 55 articles in 1999 to 363 articles in 2021. The fastest increase was observed in the period of 2004 to 2014. Saad F, 2004, *J Natl Cancer I* (16) and Roodman G, 2004, *New Engl J Med* (17) played critical roles in the research of BM in PCa (**Figure 8**).

With respect to countries, the USA leads the way in research in this field. The USA has published 1,890 related articles in the past 22 years, while the second place is occupied by China with only 535 articles. Furthermore, USA has an H-index of 142 and a total number of 98,773 citations. China, ranking second in the output, has made great achievements in the field in the past 10 years (**Figure 3B**). However, the H-index (51) and AAC (20.06 times) of China fall behind those of the USA and European countries, which demonstrates that the quality of the research needs to be continually improved. Moreover, 80% of the top 10 productive institutions come from the USA, and all top five productive institutions are American. Although the University of Texas MD Anderson Cancer Center published most articles, the University of Michigan, ranking second in the production, has the most citations (8,902). As regards the quality of articles, Massachusetts General Hospital had the highest AAC with 68 articles and a total of 8,262 citations.

Furthermore, nearly all authors with authority in the field come from the USA and the UK (**Table 3**). Of the top 10 productive authors, 70% are American and 30% come from the UK. Saad F, coming from the USA, has published the most articles (almost twice as many as the second most productive author). However, Fizazi K and Smith MR have much higher AAC values than other authors.

*Prostate*, as a specialty journal, has published the most related articles in the past 22 years. *European Urology* (EU), from the Netherlands, is the most influential journal in the field of urology. EU has published 66 related articles in this period, which also indicates that BM in PCa is a hotspot in urology. Besides this, there are also some journals with low impact factors, such as *Plos One*, having published lots of related articles.

The concept of document co-citation in co-citation analysis was first proposed by Small and Marsakova. Document co-citation means that, if two documents A and B are jointly cited by a later published document C, then A has a co-citation relationship between B, which implies that they have a topic similarity relationship. As shown in **Table 5**, most of the top co-cited references were published during the first decade of the 21st century. According to **Figure 8**, Saad F, 2002, *Jnci-J Natl Cancer I* (18) initiated the first strong citation burst in 2002. Interestingly, there was no newly published reference causing a strong citation burst from 2004 to 2009. With the publication of Fizazi K, 2009, *Journal of Clinical Oncology*, the second wave of citation burst began, and it persists to the present.

In recent years, the most frequently used author keywords were “CT”, “tumor microenvironment”, “biomarkers”, “mCRPC”, “enzalutamide”, “radium-223”, and “bone-targeted agents” (**Figure 9B**). This demonstrates that CT, which is relatively precise and cheap, is the most frequently used diagnosis modality in BM. Furthermore, “tumor microenvironment” and “biomarkers” are the two hotspots in the research of the mechanism of BM in PCa. The immune microenvironment of primary PCa is considered “cold” while BM is relatively “hot” (19). According to Galon J’s study (20), the immune microenvironment of BM is not inherently “hot”, but it can be targeted by inhibiting the CCL20–CCR6 axis, which takes part in immune suppression and a lot of inflammatory and immune-activated states, including autoimmune disease (21). Consequently, through the research of “tumor microenvironment” and “biomarkers”, finding suitable and effective therapeutic targets has a general prospect in the treatment of BM. “mCRPC” is a difficult and long-standing problem in PCa. BM is found in 90% of mCRPC patients, which can be explained by the tropism of PCa to bone (22). The first-line treatments of mCRPC include the androgen synthesis inhibitor abiraterone, the androgen receptor (AR) inhibitor “enzalutamide”, the chemotherapeutic docetaxel, and the immunotherapeutic sipuleucel-T (23). It was reported that abiraterone can improve the overall survival by 4.4 months compared with the controlled group (34.7 *vs*. 30.3 months, *p* = 0.0033) (24). Enzalutamide, a second-generation nonsteroidal AR inhibitor, affects the AR pathway in three ways: it binds to the AR with greater relative affinity than bicalutamide, it reduces the efficiency of AR nuclear translocation, and it impairs both DNA binding to androgen response elements and the recruitment of co-activators (25). According to Fizazi K and Scher HI’s research (26, 27), enzalutamide can improve the median overall survival by 4.8 months. Docetaxel is another important treatment option for mCRPC, with 2–2.9 months of improvement in median survival compared with mitoxantrone plus prednisone therapy (28, 29). A phase III trial of sipuleucel-T showed 4.1 months of improvement of survival in 512 asymptomatic or minimally symptomatic mCRPC patients compared with the placebo group (30). Bone-targeted agents, including bisphosphonates, denosumab, and radium-223, are important for BM patients because they can prevent SREs to improve the quality of life and survival. However, despite progress in the development of new drugs, mCRPC is still incurable, and more targeted research is needed. The noncurative therapy for BM just aims to prolong survival, palliate symptoms, improve and maintain the quality of life, and prevent complications. In addition, the psychological impact of PCa treatments on patients is also a matter of concern. It has been reported that psychological disease, depression, and anxiety have incidence rates of 20%–28%, 4%–19%, and 7%–32%, respectively, in patients receiving radical prostatectomy. Interestingly, the psychological impact on patients under active surveillance is much more serious than the impact on patients receiving radiotherapy (31). Actually, artificial intelligence, an emerging research field in PCa, may be able to better predict the side effects of different treatments in the future (32).

Bibliometric analysis, because of its function of summarizing research hotspots in a certain research field, can provide directions for future research in this field. Through a bibliometric analysis of prostate cancer bone metastases, we were able to summarize the research in this field over the past few decades. More importantly, we can learn about the research hotspots in this field in recent years, and researchers will benefit from it. Scientific research not only needs to keep moving forward but also needs to summarize the past and gain experience and direction from past research. Therefore, the field of prostate cancer bone metastases research would benefit from the bibliometric analysis of clinical and biomedical exploration—for example, clinical bibliometric analysis can tell us the research hotspots for clinical diagnosis or treatments of prostate cancer bone metastases. In addition, from the bibliometric analysis of biomedicine, we can learn the hotspots in the research on the mechanism of bone metastasis in prostate cancer, and researchers will be guided to pay attention to this aspect. In sum, these could advance the field of prostate cancer bone metastases research.

### Limitations

There are still some limitations in our study. First, we cannot ensure that all related articles were included with our search strategy, which may cause a bias of the results. Second, recently published articles have not had enough time to be cited.

## Conclusion

Based on the present bibliometric analysis of BM in PCa, we conclude that the research field of BM in PCa has been getting hot since 1999. USA leads the way in nearly all aspects of the research field, such as the most productive institutions and authors. Notably, mCRPC has been the hottest topic in PCa research in recent years. CT is the most common diagnostic method for BM. Enzalutamide and radium-223, as important treatment modalities for BM in PCa, have attracted widespread attention. Furthermore, studies focus on the tumor microenvironment and biomarkers to explore the mechanism and the therapeutic targets of BM in PCa. In the future, perhaps mCRPC therapies will be the most critical research field of BM in PCa.

## Data availability statement

The raw data supporting the conclusions of this article will be made available by the authors, without undue reservation.

## Author contributions

ZG, HH and YL conceived and designed the study. YC and CT analyzed the data by using Excel and wrote the manuscript. ZS and JZ visualized the data by using VOSviewer and Citespace. ZL and LL searched and downloaded the data from WoSCC. SP and WW revised and reviewed the manuscript. All authors contributed to the article and approved the submitted version.

## Funding

This study was supported by the National Natural Science Foundation of China (nos. 81772733 and 81972384) and Guangdong Scientific Research Projects (no: 2021A1515010223) to ZG, the National Natural Science Foundation for Young Scientists of China (no: 81802527) and Beijing Bethune Charitable Foundation (no: mnzl202026) to YL, Guangdong Provincial Clinical Research Center for Urological Diseases (2020B1111170006), and Guangdong Science and Technology Department (2020B1212060018), National Natural Science Foundation of China (No:81974395, No:82173036), Guangdong Basic and Applied Basic Research Foundation(No: 2019A1515011437), International Science and technology cooperation project plan of Guangdong Province (No: 2021A0505030085), Sun Yat-Sen University Clinical Research 5010 Program (No: 2019005) and Sun Yat-Sen Clinical Research Cultivating Program (No: 201702) to HH.

## Acknowledgment

We thank LetPub (www.letpub.com) for its linguistic assistance during the preparation of this manuscript.

## Conflict of interest

The authors declare that the research was conducted in the absence of any commercial or financial relationships that could be construed as a potential conflict of interest.

## Publisher’s note

All claims expressed in this article are solely those of the authors and do not necessarily represent those of their affiliated organizations, or those of the publisher, the editors and the reviewers. Any product that may be evaluated in this article, or claim that may be made by its manufacturer, is not guaranteed or endorsed by the publisher.
